# MicroRNA 195-5p Targets *Foxo3* Promoter Region to Regulate Its Expression in Granulosa Cells

**DOI:** 10.3390/ijms22136721

**Published:** 2021-06-23

**Authors:** Yinshan Bai, Bo Pan, Xiaoshu Zhan, Hailey Silver, Julang Li

**Affiliations:** 1 Department of Life Science and Engineering, Foshan University, Foshan 528225, China; baiyinshan@fosu.edu.cn (Y.B.); xzhan01@uoguelph.ca (X.Z.); 2 Department of Animal BioSciences, University of Guelph, Guelph, ON N1G 2W1, Canada; bopan@uoguelph.ca (B.P.); hsilver@uoguelph.ca (H.S.); 3 Cell and Developmental Biology Center, Laboratory of Chromosome Dynamics and Evolution, National Heart, Lung, and Blood Institute, National Institute of Health, Bethesda, MD 20892, USA

**Keywords:** miRNA, nuclear, granulosa, cell nuclear, *Foxo3*, transcription, mRNA, epigenetic regulation AGO

## Abstract

Forkhead box O3 (Foxo3) is a member of the FOXO subfamily within the forkhead box (FOX) family, which has been shown to be essential for ovarian follicular development and maturation. Previous studies have shown the abundant expression of miR-195-5p in the nuclei of porcine granulosa cells (GCs), suggesting its potential role during ovarian follicle growth. In this study, a conditional immortalized porcine granulosa cell (CIPGC) line was used to determine whether the expression of Foxo3 could be regulated by the nuclear-enriched miR-195-5p. Through silico target prediction, we identified a potential binding site of miR-195-5p within the *Foxo3* promoter. The over-expression of miR-195-5p increased Foxo3 expression at both mRNA and protein levels, while the knockdown of miR-195-5p decreased the expression of Foxo3. Furthermore, driven by the *Foxo3* promoter, luciferase reporter activity was increased in response to miR-195-5p, while the mutation of the miR-195-5p binding site in the promoter region abolished this effect. In addition, the siRNA knockdown of Argonaute (AGO) 2, but not AGO1, significantly decreased *Foxo3* transcript level. However, miR-195-5p failed to upregulate *Foxo3* expression when AGO2 was knocked down. Moreover, chromatin immunoprecipitation (CHIP) assay showed that anti-AGO2 antibody pulled down both AGO2 and the *Foxo3* promoter sequence, suggesting that AGO2 may be required for miR-195-5p to regulate *Foxo3* expression in the nucleus. Additionally, *Foxo3* expression was significantly increased by valproic acid (VPA), the inhibitor of deacetylase, as well as by methyltransferase inhibitor BIX-01294, indicating the involvement of histone modification. These effects were further enhanced in the presence of miR-195-5p and were decreased when miR-195-5p was knocked down. Overall, our results suggest that nuclear-enriched miR-195-5p regulates *Foxo3* expression, which may be associated with AGO2 recruitment, as well as histone demethylation and acetylation in ovarian granulosa cells.

## 1. Introduction

MicroRNA (miRNA) have been traditionally known to post-transcriptionally silence gene expression in the cytoplasm via binding to the 3′-UTR of mRNA [1]. More recently, it has been revealed that some miRNAs are enriched in the cell nucleus, where they regulate gene expression at the transcriptional level [2,3]. Specifically, Argonaute (AGO) 2 binds to and unwinds the double-stranded small RNA in the cytoplasm, creating mature miRNA [4]. The miRNA-AGO2 complex may subsequently be transported into the nucleus via Importin 8 (IPO8), a protein belonging to the karyopherin β group. IPO8-assisted transport depends on its physical association and interaction with the AGO protein [3]. The nuclear RNA-induced silencing complex (RISC) is then formed in the nucleus, where the miRNA can act to modify gene expression in different ways, such as through epigenetic modifications, transcriptional gene expression regulation, and influencing the expression of other miRNA (reviewed by Liu and colleagues) [4]. In addition to AGO2, the trinucleotide repeat-containing 6 (TNR6, also known as GW182) has also been identified as a key component of RISC. The size of the nuclear RISC is only approximately 158 kDa, which is almost 20-folds smaller than cytoplasmic RISC (nearly 3 MDa) [4].

Ovarian follicular development is a highly orchestrated process that is regulated by endocrine and autocrine factors. It is increasingly evident that the somatic components of the ovary have a complex network of miRNAs that fine-tune its cellular activity [5,6,7], such as those involved in follicular development and maturation [8,9,10,11]. Further, it is known that over 99% of ovarian follicles undergo atresia via apoptotic programmed cell death [12]. Understanding the mechanism behind the precise regulation of follicle growth and ovulation, compared to atresia, is highly essential to improve fertility. Although the functions of some nuclear miRNA have been found in cancerous and other cell lines [13,14], the role of nuclear miRNA in regulating ovarian cell gene expression remains unknown.

Forkhead box O (FOXO) is a transcription factor family expressed in various cell types that control diverse cellular activities, such as cell proliferation, metabolism, and apoptosis [15,16,17]. FOXOs are not only able to regulate the expression of genes that influence the cells in which they are expressed, but they are also able to regulate the expression of genes whose products serve as endocrine or metabolic regulators for tissue development [18]. It is well established that *Foxo3* plays an important role in ovarian follicular development and maturation [19]. Importantly, Foxo3 expression and other members of the FOXO family are known to be heavily regulated by multiple microRNAs and proteins [20]. However, there is a need to further understand the mechanisms behind its expression and the specific functions miRNA may execute in this process.

We previously performed an ovarian granulosa cell (GC) transcriptome analysis on the nuclear and cytoplasmic fractions, respectively. This revealed that miR-195-5p is enriched in the nucleus, suggesting the potential nuclear-specific function of this miRNA during ovarian follicle growth [21]. The objective of the current study was to investigate the potential role of miR-195-5p acting in the nucleus of ovarian GCs. It was found that miR-195-5p upregulated *Foxo3* at the transcript level, possibly via epigenetic modification at its promoter region.

## 2. Materials and Methods

### 2.1. MiRNA Target Prediction

Using the computational prediction software MicroPIR2 (https://tools4mirs.org/software/mirna_databases/micropir2/, accessed on 17 April 2021) [22], we searched for potential gene promoters that may be targeted by miR-195-5p. This software contains a database with over 80 million miRNA predicted targets in promoter sequences of the human genome. miRNA targets were searched by miRNA name with the following criteria: (1) an average *p*-value ≤ 0.05; (2) an average conservation score ≥ 0.85; (3) a maximum number of four unpaired nucleotides; and (4) near perfect matching in the seed region. After the human target sequences of miR-195-5p were selected, we used the “BLAST” feature in Ensembl (http://asia.ensembl.org/index.html, accessed on 17 April 2021), a genetic database, to determine if the predicted human promoter sequences are conserved in *Sus Scrofa*. The selected genes were then searched in the Ensembl *Sus scrofa* database to determine the locations of the selected target sequence relative to the start codon, ATG, in order to confirm the target sequence is indeed in the promoter region. The promoter region is defined as ~1000 bp upstream of ATG and ~200 bp downstream of ATG [23]. Furthermore, miRNA primers were designed based on human sequence that were also compatible with *Sus scrofa*, and were ordered from EXIQON (Exiqon, Vedbaek, Denmark). The primers were developed for the target sequence of miR-195-5p using the Primer-BLAST feature in NCBI and were ordered from Integrated DNA Technologies (IDT, Coralville, IA, USA).

### 2.2. Cell Culture and Cell Counting

The conditional immortalized porcine granulosa cell (CIPGC) line constructed by Bai et al. [22] was used as our cell model in the present study. This cell line allows for the long-term observation of primary porcine GCs [24], as differentiation is suppressed when cultured with doxycycline (Dox, Sigma, Cat#: D9891, Burlington, MA, USA). As an immortalized cell line, CIPGCs can steadily propagate for at least half a year and display the same cell morphology and estradiol production pattern as primary GCs. The CIPGCs were cultured in DMEM/F12 basic medium (WISENT, Quebec, QC, Canada) with 10% fetal bovine serum (FBS) (WISENT, Cat#: 080-450, Quebec, QC, Canada), 1% GlutaMAX (100×, Gibco, Cat#: 35050061, Waltham, MA, USA), 0.02% puromycin and 0.2% of Dox. The CIPGCs were plated on a 6-well plate with approximately 2000 cells per each well and were incubated at 38 °C with 5% CO_2_ concentration. The following day, diluted miR-195-5p mimics and inhibitors were transfected into respective wells using transfection reagent (Attractene Transfection, Qiagen, Cat#: 301005, Hilden, Germany). After incubating for 6 h, the culture medium was changed. Following an additional 48 h, cells were collected using Trypsin, and were counted using Bio-Rad TC20 Cell Counter after staining with Trypan Blue.

### 2.3. Nuclear Separation

In order to examine miRNA expression independently in the nucleus and the cytoplasm, the nuclear and cytoplasmic components of the CIPGCs were separated using the Nuclei Isolation Kit (Sigma, Cat#: NUC101, Burlington, MA, USA). The nuclei yield and purity were tested through using the Bio-Rad TC20 Cell Counter and through microscopic examination via staining in Hochest33342, respectively.

### 2.4. Western Blot

To confirm the clean separation of the nuclear and cytoplasmic components, the respective extracts were assessed for protein presence and concentration using the DC Protein Assay Kit (Bio-Rad, Cat#: 5000111, Berkeley, CA, USA). Western blots were performed as previously described [10], and CIPGC samples were used as a positive control. Total protein was lysed using RIPA buffer to extract nuclear, cytoplasmic, and cellular components, respectively. The samples were electrophoresed on 11% sodium-dodecyl sulfate polyacrylamide (SDS-PAGE) gel. The primary antibodies anti-Lamin B (1:10,000 dilution, Abcam, Cat#: ab16048 Cambridge, MA, USA) and anti-GAPDH (1:5000 dilution, Santa Cruz, Cat#: sc-137179, Dallas, TX, USA) were used to test the purity of both the cytoplasmic and nuclear samples, respectively. Anti-mouse IgG (1:5000 dilution, HRP-linked; Cell Signaling Technology; Cat#: 7076, Danvers, MA, USA) was used to detect anti-GAPDH, and anti-rabbit IgG (1:10,000 dilution, HRP-linked; Cell Signaling Technology; Cat#: 7074S, Danvers, MA, USA) was used to detect anti-LAMIN B. Anti-FOXO3 (Abcam, Cat#: ab12162, Cambridge, UK) primary antibody was used to test the expression level of FOXO3. The membrane was imaged using Image Lab Software (Version 6.1, Bio-Rad, Berkeley, CA, USA). Each experiment was performed in triplicate.

### 2.5. RNA Isolation

RNA was isolated from CIPGCs, as well as its nuclear and cytoplasmic fractions, using the Total RNA Purification Kit (NORGEN, Cat#: 17200, Thorold, ON, CA, USA) and following the provided protocol. Each lysate was prepared by combining with 350 µL Buffer RL and 200 µL of 100% ethanol. Up to 600 µL of lysate and solution was applied to each assembled spin column and centrifuged for 1 min at 6000 rpm. Then, 400 µL of Wash Solution was applied and centrifuged at 14,000 rpm for 2 min, and the produced flowthrough was discarded. Each tube underwent an on-column DNA removal, in which a mixture of 7.5 µL of DNase I and 100 µL of Enzyme Incubation Buffer was applied per tube and spun at 14,000 rpm for 1 min. The flowthrough was pipetted to the top of the column and centrifugation was repeated. Washing with Wash Solution as described previously was then repeated twice, and the column was placed in a fresh 1.7 mL tub. Subsequently, 50 µL of Elution Solution A was applied, and the column was spun at 2000 rpm for 2 min, followed by 1 min at 14,000 rpm. The concentration and purity of the isolated RNA were tested using NanoDrop8000 (Thermo Fisher, Cat#: ND8000-GL, Waltham, MA, USA) in order to ensure the samples were viable for reverse transcription.

### 2.6. MiRNA Reverse Transcription, cDNA Synthesis, q-PCR

The miRNA was reverse transcribed, synthesized into complementary DNA (cDNA), and prepared for quantitative-PCR (q-PCR) using the miRCURY kit (Exiqon, Cat#: 203400, Vedbaek, Denmark), following its protocols. Briefly, RNA samples were diluted to a concentration of 5 ng/µL, and 1 µL of synthetic spike was added per 20 ng of RNA. The reverse transcription reaction was set up for a total volume of 10 µL per RNA sample. A thermocycler was used to incubate the sample at 42 °C for 60 min, followed by heat inactivation at 95 °C for 5 min, and subsequent cooling to 4 °C. The miRNA cDNA was stored at −20 °C until analysis.

The miRNA cDNA was adjusted to a 30× dilution with nuclease-free water to prepare for q-PCR. Although 80× dilution is recommended by the manufacturer, this dilution had the highest success rate in our labs, as determined experimentally. 5 µL of PCR Master mix was combined with 4 µL of diluted cDNA and 1 µL of miRNA primer mix and was mixed thoroughly and quickly centrifuged. Samples were amplified via q-PCR, beginning with 95 °C for 10 min, followed by 45 amplifications cycles at 95 °C and 1 min at 60 °C, with a ramp-rate of 1.6 °C/s^4^.

### 2.7. Target Gene cDNA Synthesis and q-PCR

Prior to cDNA synthesis, target gene primers underwent a primer efficiency test to ensure they were viable for q-PCR testing. MasterMix solution, comprising of 7.5 µL of SYBR, 2.2 µL of sterile H_2_O, and 0.3 µL of both the forward and reverse primers, was added to that primer’s respective section in the 96-well plate. The primers were amplified for 40 cycles via q-PCR, and efficiencies between 90–110% were considered viable. cDNA for target gene samples were synthesized using the Applied Biological Materials 5× All-In-One RT MasterMix kit (Abm, Cat#: G490, New York, NY, USA) and following its protocol. Briefly, 4 µL of MasterMix was added to RNA samples that had been diluted with nuclease-free water to a concentration of 1 µg per 20 µL. Reagents were mixed well and briefly spun down. Subsequently, the thermocycler was used to synthesize the target gene cDNA, in which samples were incubated to 42 °C for 50 min, heated to 85 °C for 5 min and then cooled to 4 °C. cDNA was stored in −20 °C conditions.

### 2.8. Target Gene Expression with Mimics and Inhibitors

In order to measure target gene expression via qPCR, the cDNA was first diluted 12×. 5 µL of Bio-Rad’s SYBR was combined with 3 µL of diluted cDNA and 1.6 µL of water. This was mixed with 0.2 µL of both the forward and reverse primers for *Foxo3*, for a total reaction volume of 10 µL. The samples were then amplified via Real-time quantitative PCR (qPCR).

### 2.9. Chromatin Immunoprecipitation

Chromatin immunoprecipitation (ChIP) assay was performed according to the instructions of Pierce Agarose ChIP Kit (Thermo Scientific, Cat#: PI26156, Waltham, MA, USA), using antibodies for AGO2 (Abcam, Cat#: ab32381, Cambridge, UK). The cell lysates were treated with both protease and phosphatase inhibitors prior to immunoprecipitation. DNA was sonicated into fragments of 200–700 bp. Real-time quantitative PCR (qPCR) was performed with SYBR Green PCR Master Mix and primer sets targeting the binding site of miR-195-5p within the *Foxo3* promoter (Table S1). The amount of immunoprecipitated DNA was calculated in reference to a standard curve and normalized to input DNA.

### 2.10. Dual-Luciferase Reporter Vectors

The wild type and mutant promoter of *Foxo3* were cloned into the pGL3-promoter Dual-Luciferase reporter vector (Promega, Madison, WI, USA) to validate the relationship between miR-195-5p and *Foxo3*. The wild type contained the putative miR-195-5p binding sites of 5′-UGUCUAUAACUUUGUGCUGCUGC-3′. The mutant sequence of the binding sites was replaced by 5′-AGACAAAAUCAUAGAGGUCCAGG-3′. The sequence of Renilla luciferase was constructed in a plasmid vector as a reporter fluorescence, and firefly luciferase was inserted as an internal reference. Prior to transfection, 293FT cells were seeded into 24-well plates (2 × 10^4^ cells/well). Then, 25 ng of wild type or mutant vector was co-transfected into the cells, along with 100 nM of miR-195-5p mimics, inhibitor, or negative control. After 48 h, luciferase activity was detected using the Dual-Glo luciferase reporter assay system (Promega, Cat#: E1910, Madison, WI, USA).

### 2.11. Statistical Analysis

Data were analyzed by one-way ANOVA via SPSS 13.0 Software (SPSS Inc., Chicago, IL, USA). Results were presented as means ± SEM. Duncan’s multiple-range test was performed if differences were identified between the groups. Differences were considered statistically significant at *p* < 0.05.

## 3. Results

**Figure 1:**
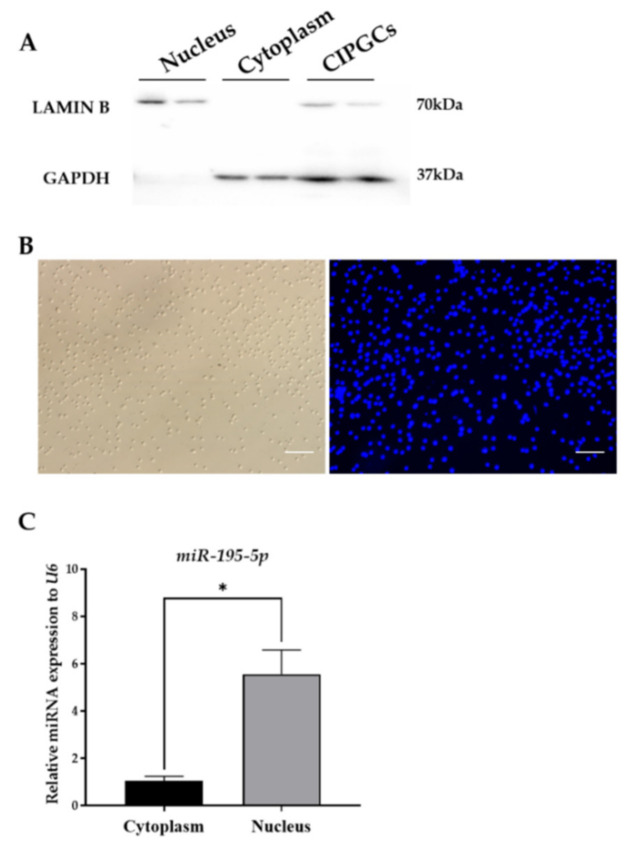
miR-195-5p is highly expressed in the nucleus of conditional immortalized porcine granulosa cell (CIPGCs) line. (**A**) Western blot assay showing the quality of the cytoplasmic and nuclear components isolated from CIPGCs. (**B**) Hoechst 33,342 staining identifying the nuclei isolated from CIPGCs (scale bar 100 µm). (**C**) RT-qPCR showing miR-195-5p levels in both cytoplasmic and nuclear components isolated from CIPGCs. Data are presented as mean ± SEM of three repeated experiments (n = 6 per group each time). * indicates *p* < 0.05 when compared with control.

We previously found that miR-195-5p is enriched in the nucleus of primary GCs, compared to the cytoplasm. In this study, we first sought to verify this finding in a conditional immortalized porcine granulosa cell (CIPGC) line. Nuclear and cytoplasmic components were isolated from CIPGCs, and the separation of the two components was confirmed via Western blot analysis, using anti-LAMIN B and anti-GAPDH antibodies to target nuclear and cytoplasmic-specific proteins, respectively (Figure 1A). The quality of the nuclei after isolation and purification was confirmed with Hoechst 33,342 staining (Figure 1B). As shown in Figure 1C, a more than five-fold enrichment of miR-195-5p was observed in the nucleus compared to the cytoplasm.

**Figure 2:**
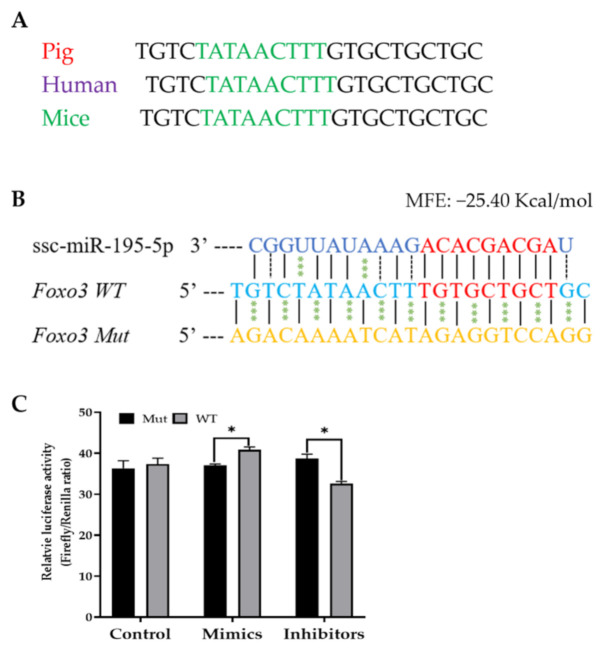
miR-195-5p transcriptionally regulates the expression of the *Foxo3* gene via binding to its promoter region. (**A**) Comparison of miR-195-5p binding sites among pig, human and mice species. The TATA Box is highlighted in green. (**B**) The predicted binding site of miR-195-5p within the *Foxo3* promoter and the mutated sequence of the predicted binding site for the luciferase. The seed sequence of miR-195-5p and the predicted binding site on the *Foxo3* promoter are highlighted in red. (**C**) Dual-Luciferase assay was performed on the 293FT cell line. miRNA negative control, as well as miR-195-5p mimics and inhibitors were co-transfected with wild-type (WT) or mutant (Mut) luciferase vectors, respectively. Data are means ± SEM of three independent experiments. * indicates *p* < 0.05 when compared with control.

Using MicroPIR2, a computational prediction software that contains a database of miRNA predicted promoter sequence targets in the human genome, we next searched for gene promoters that may be targeted by miR-195-5p. MiRNA targets were searched by miRNA name, specifying for results with an average *p*-value ≤ 0.05, an average conservation score ≥ 0.85, and a maximum number of four unpaired nucleotides. The binding pattern and sequence were observed with consideration towards near-perfect matching in the seed region. After the human targets for miR-195-5p were selected, we then used the “BLAST” feature in Ensembl, a genetic database, to determine if the predicted human promoter target sequence is conserved in *Sus scrofa*. We next used the Ensembl *Sus scrofa* database to search for the selected genes in order to confirm the target sequences are located in the promoter region [23]. According to these selection criteria, a potential target of miR-195-5p was identified on the *Foxo3* promoter. Sequence alignment of the predicted binding sites in the promoter region across pig, human, and mice species revealed a 100% homology (Figure 2A), suggesting that this site is evolutionarily conserved. We hypothesized that miR-195-5p interacts with the *Foxo3* promoter and affects its transcription in the nucleus. To test this hypothesis, the *Foxo3* promoter with the wild type and mutated binding sites were inserted into a firefly luciferase reporter vector (Figure 2B), respectively, and dual-luciferase activities were analyzed. It was found that the wild-type promoter activity of *Foxo3* was increased when transfected with the miR-195-5p mimics, and it was reversed when transfected with the miR-195-5p inhibitors (Figure 2C).

**Figure 3:**
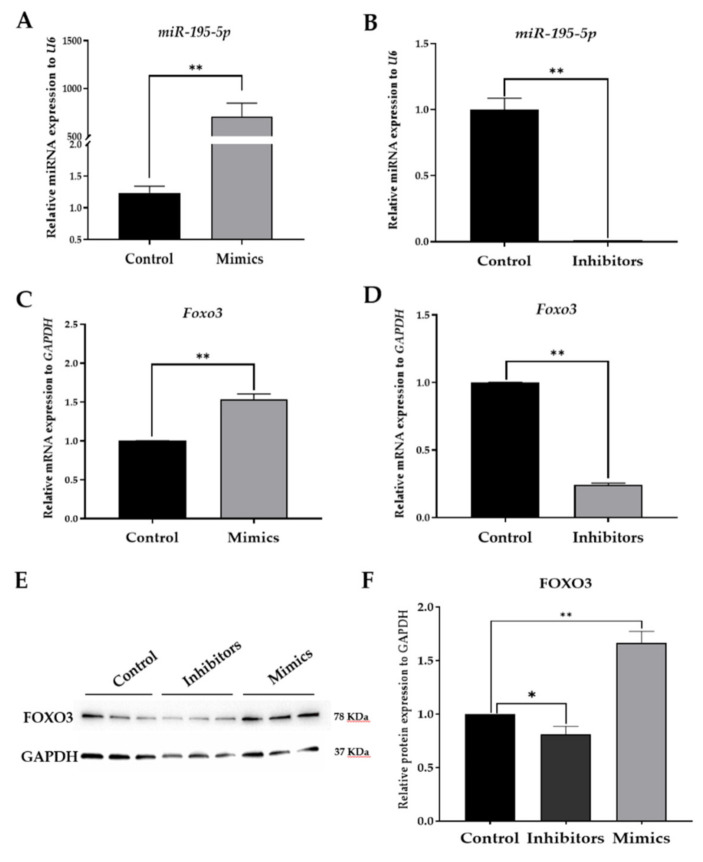
miR-195-5p promotes transcription of *Foxo3* in CPIGCs. (**A**,**B**) RT-qPCR showing the transfection efficiency of miR-195-5p mimics and inhibitors transfected into CPIGCs for 24 h. (**C**,**D**) RT-qPCR indicating the mRNA expression level of *Foxo3* in CPIGCs transfected with miR-195-5p mimics and inhibitors, respectively. (**E**,**F**) Western blot assay showing the protein expression level of FOXO3 in CPIGCs after transfection with a miRNA negative control, as well as miR-195-5p inhibitors and mimics, respectively. Data are mean ± SEM of three independent experiments. * indicates *p* < 0.05, and ** indicates *p* < 0.01 when compared with control.

To further verify this prediction, miR-195-5p over-expression and knock down experiments were performed. Figure 3A,B shows that the intracellular levels of miR-195-5p can be effectively manipulated using its mimics and inhibitors, respectively. As shown in Figure 3C,D, *Foxo3* expression was significantly up-regulated by miR-195-5p mimics and down-regulated by its inhibitors. The same response by FOXO3 was also observed at the protein level when examined via Western blot analysis (Figure 3E,F), confirming that its expression is regulated by miR-195-5p.

**Figure 4:**
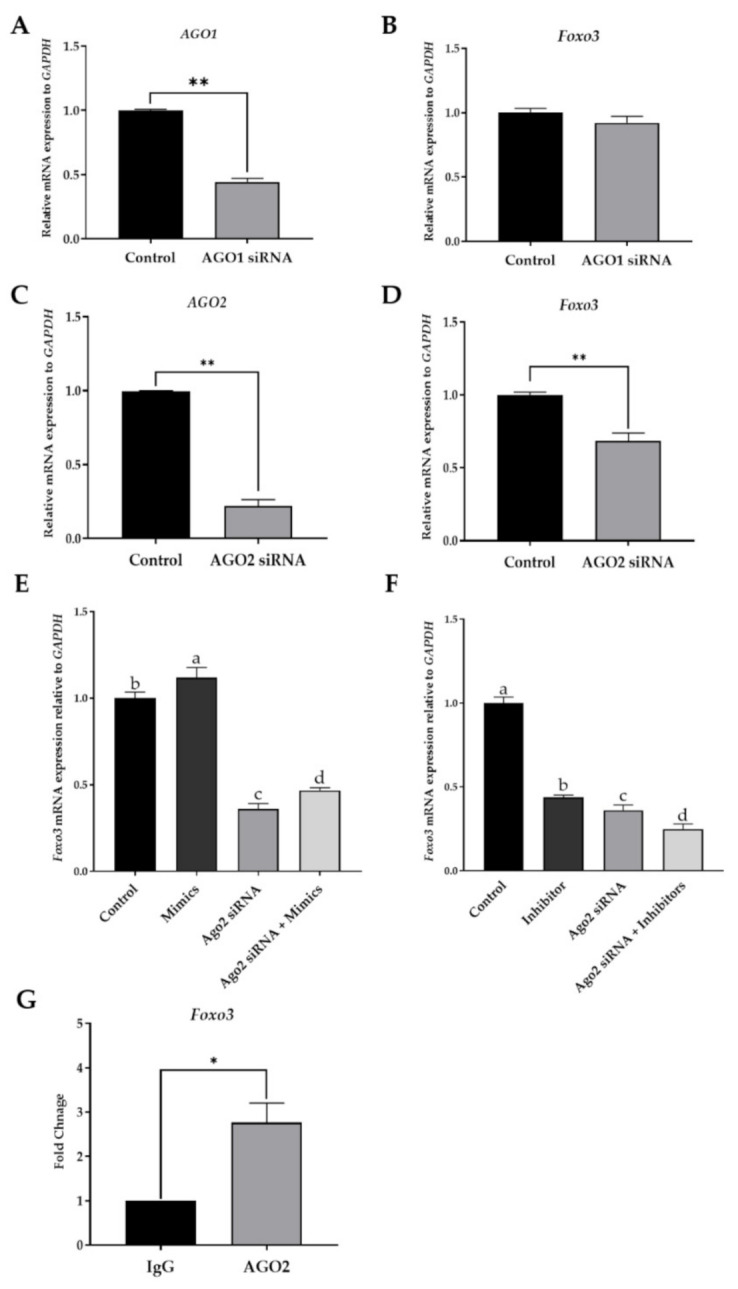
miR-195-5p promotes transcription of *Foxo3* in CPIGCs in an AGO2-dependent manner. (**A**–**D**) SiRNA knockdown assay indicating the *Foxo3* transcription in CPIGCs transfected with AGO1 siRNA and AGO2 siRNA. (**E**) RT-qPCR showing the mRNA level of *Foxo3* in CPIGCs transfected with AGO2 siRNA and miR-195-5p mimics, respectively or together. (**F**) *Foxo3* expression regulated by AGO2 and miR-195-5p inhibitor. (**G**) CHIP and RT-qPCR showing the enrichment of *Foxo3* promoter in the AGO2 complex. Data are mean ± SEM of three independent experiments. * indicates *p* < 0.05, and ** indicates *p* ≤ 0.01 when compared with control. Means that were assigned different letters (a, b, c, d) were found to be significantly different (*p* < 0.05).

To study if AGO proteins are involved in the regulation of *Foxo3* expression, a knockdown study was performed using siRNAs against AGO1 and AGO2, respectively. It was found that the respective siRNA efficiently down-regulated its associated AGO mRNAs in the CIPGC line (Figure 4A,C). AGO1 knockdown had no significant effect on *Foxo3* expression (Figure 4B). However, at the transcript level, *Foxo3* was significantly decreased when AGO2 was knocked down (Figure 4D), suggesting that it has a role in *Foxo3* expression regulation. To further investigate the function of AGO2 in miR-195-5p-induced *Foxo3* expression, CIPGCs were cultured in the absence or presence of miR-195-5p mimic, and in siRNA against AGO2, respectively or together. As shown in Figure 4E, miR-195-5p was unable to upregulate *Foxo3* expression in the presence of siRNA against AGO2 compared to the control group. Moreover, the *Foxo3* expression in CIPGCs were significantly decreased when treated with siRNA against AGO2 and/or miR-195-5p inhibitor (Figure 4F), suggesting that the regulation by miR-195-5p is mediated by AGO2. To further investigate whether AGO2 may bind to the *Foxo3* promoter, we performed a chromatin immunoprecipitation (CHIP) assay in CIPGCs. In chromatin fractions pulled down by anti-AGO2 antibody, the level of the *Foxo3* promoter sequence was significantly higher compared to those pulled down with an IgG control. This finding suggests that AGO2 complexes with the *Foxo3* promoter DNA (Figure 4G). Taken together, the results from the two complementary experiments AGO2 depletion and CHIP support the notion that AGO2 is required for miR-195-5p to regulate *Foxo3* expression in the nucleus.

**Figure 5:**
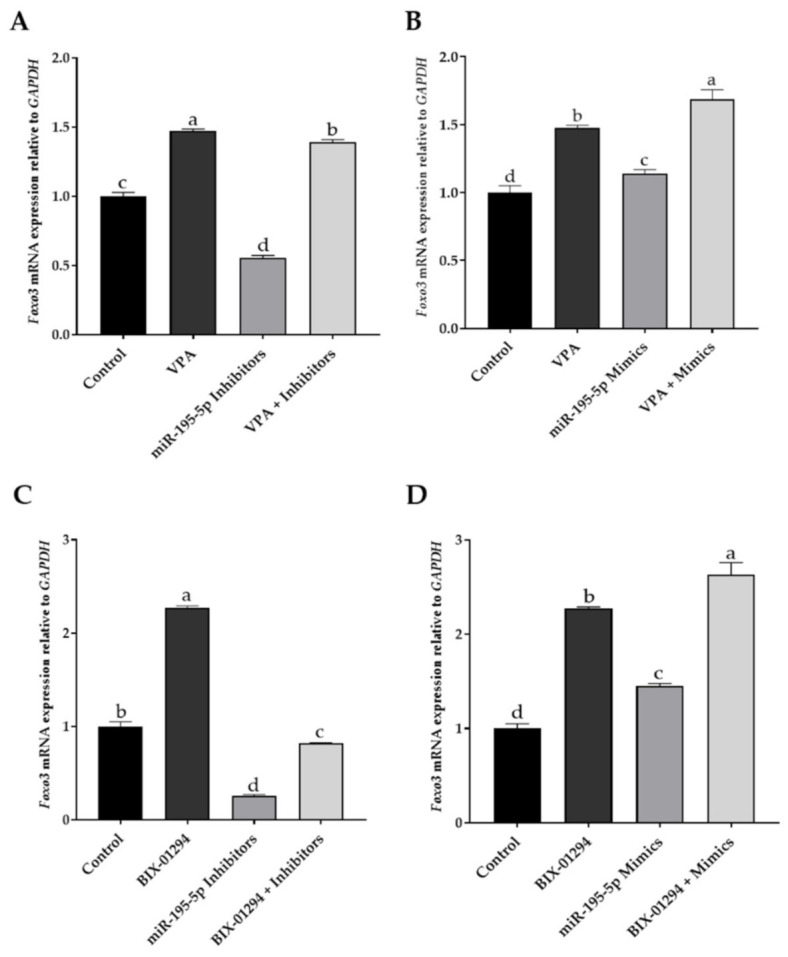
miR-195-5p demonstrates histone modification involved in *Foxo3* expression. (**A**,**C**) The inhibition effect of miR-195-5p on *Foxo3* expression was reversed by the specific inhibitor histone deacetylases-Valproic acid (VPA), and by the inhibitor G9a histone methyltransferase-BIX-1294, respectively. (**B**,**D**): The stimulating effect of miR-195-5p mimics on *Foxo3* expression was enhanced by the addition of VPA and BIX-1294. Data are mean ± SEM of three independent experiments (n = 3). Means that were assigned different letters (a, b, c, d) were found to be significantly different (*p* < 0.01).

**Figure 6:**
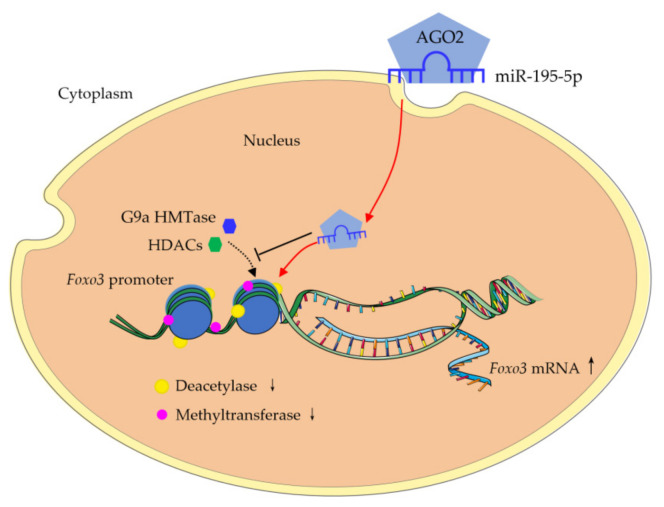
Model of AGO2-miR-195-5p complex assembly in the *Foxo3* promoter region, which leads to the epigenetic modifications that regulate the expression of *Foxo3*. AGO2: Argonaute 2. G9a HMTase: G9a histone methyltransferase; HDACs: histone deacetylases. ↑ means up-regulation, ↓ means down-regulation.

Methylation and histone acetylation are associated with transcription regulation. Valproic acid (VPA) is a specific inhibitor of histone deacetylases (HDACs), and BIX-1294 is an inhibitor of G9a histone methyltransferase (G9a HMTase). If histone acetylation and hypomethylation are downstream of miR-195-5p activities, an inhibitor of HDACs and histone methyltransferase would reverse the suppression effect on *Foxo3* expression caused by siRNA knockdown of miR-195-5p. As shown in Figure 5A,C, the suppression of *Foxo3* expression by miR-195-5p knock-down via using an inhibitor was reversed by VPA and BIX-1294, respectively. On the other hand, the stimulation effect of miR-195-5p mimic on *Foxo3* expression was further enhanced by the addition of VPA and BIX-1294, respectively (Figure 5B,D). These results suggest that the involvement of miR-195-5p in *Foxo3* expression regulation may be upstream of histone acetylation and methylation (Figure 6).

## 4. Discussion

It is known that *Foxo3* is a downstream effector of the PI3K/Akt pathway. In rodents, *Foxo3* exerts its transcriptional activity in the nucleus to suppress follicular growth in quiescent primordial follicles [25]. Upon phosphorylation, *Foxo3* is translocated to the cytoplasm, where it induces follicle activation. A previous study by Castrillon et al. reported that *Foxo3* knockout mice exhibited premature primordial follicle activation and subsequent primary ovarian insufficiency (POI) by 12 weeks of age [17]. Using a conditional oocyte-specific knockout approach, the role of intraoocyte *Foxo3* in primordial follicle activation was confirmed [19]. In addition to the oocyte, *Foxo3* is expressed in the ovarian granulosa cell. The granulosa-specific deletion of *Foxo1/3* resulted in the altered expression of specific genes associated with follicle growth compared to apoptosis and infertility [26]. Moreover, altered *Foxo3* expression was associated with apoptosis in the GCs of women with polycystic ovary syndrome [27]. These findings suggest that *Foxo3* plays an important role in ovarian follicular development and maturation.

The nuclear localization and role of miRNA have been attracting attention in recent years. Abundant nuclear miRNAs have been found in various tissues and cell types, including HeLa cells [28], human Nasopharyngeal carcinoma cell lines [29], and rat myoblasts [30]. The regulating function of miRNAs in the nucleus is mainly achieved by the transcriptional activation or inhibition of genes through the mediation of epigenetic modifications, such as the methylation of H3K9 and acetylation of H3K4 [4,31]. However, the function of nuclear miRNA in GCs has not yet been described. The novelty of our study demonstrated that miR-195-5p regulates the expression of *Foxo3* in the nucleus of porcine ovarian GCs. Specifically, miR-195-5p mimic transfection resulted in an increased expression of *Foxo3*, while knockdown of miR-195-5p decreased *Foxo3* expression. Further, Luciferase reporter assay results confirmed the increased luciferase activity of the *Foxo3* promoter in response to miR-195-5p and the abolished response when the miR-195-5p binding site on the *Foxo3* promoter was mutated. Furthermore, we found that AGO2 plays critical role in this regulation process, as AGO2 knock-down suppressed miR-195-5p-induced *Foxo3* expression. AGO2 is a protein found in the cell nuclei of yeast, plants, and animals. Its role in mediating small RNA functioning has been observed to occur in mammalian nuclei [32]. For example, AGO2 was reported to facilitate RNA-mediated modulation of transcription [33,34] or splicing [35]. In the present study, the CHIP assay consistently demonstrated that AGO2 complexes with the *Foxo3* promoter in the nucleus of ovarian GCs. As it is known that Foxo3 plays an important regulatory role in follicular development and atresia [36], our results confirmed that nuclear-enriched mir-195-5p may control the expression of *Foxo3* in GCs in order to regulate follicular development and atresia.

Three different models of miRNA-promoter interaction have been described in literature, all of which include association with the AGO protein. In the DNA-RNA hybrid model, which occurs when the promoter is in an open configuration, the nuclear miR-RISC targets the TATA box motif or the transcription factor binding sites. This binding then leads to the recruitment of transcription factors or epigenetic histone modifiers [37], which subsequently activates RNA polymerase II. Its activation initiates transcription or epigenetic modification, resulting in the regulation of transcription [4]. The second model involves RNA-RNA interaction, where nuclear miRNA binds in a canonical Watson-Crick based fashion to either non-coding sense or antisense transcripts that overlap gene promoter regions [38]. Once bound, they serve as a scaffold to recruit histone modifiers, RNA polymerase, or transcription factors to transcriptionally regulate gene expression. The final model is the miR-DNA-DNA triplex, which is stabilized by the AGO protein. This complex can be formed when the promoter is in an open conformation, as it grants the miRNA access [13]. Transcription is regulated by disrupting the pre-initiation complex through altering the topography of the chromatin, either permitting or restricting transcription factor access to the promoter [4,38]. In this study, we predicted that the interaction of miR-195-5p with the *Foxo3* promoter region likely occurs through the first model; the DNA-RNA hybrid. Supporting this hypothesis is the location of the predicted miR-195-5p binding site in the TATA box of the *Foxo3* promoter, as well as the results of the dual-luciferase assay described above.

A previous study showed that AGO2, one of four AGO proteins (AGO1–4), has a role in nucleic-cytoplasmic shuttling [39]. A study by Janowski et al. showed that both AGO1 and AGO2 are necessary for the inhibition of gene expression by duplex RNAs complementary to either promoter DNA or mRNA [40]. Other studies have reported that in human neuroblastoma, fibroblast and breast cancer cells, AGO2 can shuttle microRNA from the cytoplasm into the nucleus in order to bind to the targeted gene promoter region as an AGO2-microRNA complex [41,42]. Consistent with these reports, our data suggests that the AGO protein plays a role in mediating miRNA regulation of transcription. Specifically, this study demonstrated the involvement of AGO2, as opposed to other AGO proteins, in regulating the effect of miR-195-5p on *Foxo3* transcription.

Furthermore, it is known that histone acetylation can loosen the interaction between the positively charged histone and the negatively charged DNA through making the histone more neutral [43]. However, histone methylation exerts its gene modification effect by creating a docking site for chromatin-associated proteins, which contain specific methyl histone-binding domains, and can also influence DNA methylation [44]. Several studies have shown that methylation at the promoter is typically associated with transcriptional inhibition [45,46]. Xiang et al. demonstrated that miR-584-5p induced epigenetic inactivation by the deposition of inhibition chromatin markers, such as EZH2, H3K27me3 and H3K9me2, at the gene promoter suppressed the expression of MMP-14 in human neuroblastoma [45,46]. Our findings suggest that the involvement of miR-195-5p in *Foxo3* transcription may be upstream of histone acetylation and hypomethylation. Collectively, in this study we have described the role of nuclear-enriched miR-195-5p in regulating the expression of *Foxo3* in ovarian follicular development, which may be associated with AGO2 recruitment and histone modification. However, further investigation is warranted in order to understand the mechanisms behind its involvement in transcription regulation, as it may potentially offer new insights for therapeutic intervention to improve fertility. Understanding how miRNAs are involved in gene transcription opens a door, revealing a novel layer of gene expression regulation.

## Supplementary Materials

The following are available online at https://www.mdpi.com/article/10.3390/ijms22136721/s1, Table S1: Sequences information of RT-qPCR Primers.
