# Supplementary material for: MicroRNA 195-5p Targets Foxo3 Promoter Region to Regulate Its Expression in Granulosa Cells
Source: Int J Mol Sci. 2021 Jun 23;22(13):6721. doi: 10.3390/ijms22136721 (PMC8267755; doi:10.3390/ijms22136721)
Supplement: Supplementary file 1 [file ijms-22-06721-s001.zip › ijms-1207036-supplementary.pdf]

**Table S1** Sequences information of RT-qPCR Primers

| Name | Product Size (bp) | Sequence |
|---|---|---|
| *Foxo3* IP Control | 196 | F: 5′-AAGGGGAACCGGACACCA-3′<br>R: 5′-CCGCGAGCCTCAAAGAAAAC-3′ |
| *Foxo3* IP | 171 | F: 5′-GCGCCGTGACACCCAA-3′<br>R: 5′-TATAGACACACGCAGGGTGC-3′ |
| *Ago1* | 170 | F: 5′-TAGACCCCTCACACTGGACT-3′<br>R: 5′-GATTGGTTTCCCCACAGTGC-3′ |
| *Ago2* | 216 | F: 5′-AGTGCCGAAGGAAGCCATAC-3′<br>R: 5′-ATGTTCGATGCTGGCTGTCA-3′ |
| *Foxo3* | 198 | F: 5′-ACATGGCCGGAACCATGAAT-3′<br>R: 5′-GTCCAAACACTGTGCTGCTG-3′ |
| *GAPDH* | 147 | F: 5′-TCGGAGTGAACGGATTTGGC-3′<br>R: 5′-TGCCGTGGGTGGAATCATAC-3′ |
